# Survival benefit of immune checkpoint inhibitors according to the histology in non-small-cell lung cancer: A meta-analysis and review

**DOI:** 10.18632/oncotarget.17213

**Published:** 2017-04-19

**Authors:** Bum Jun Kim, Jung Han Kim, Hyeong Su Kim

**Affiliations:** ^1^ Division of Hemato-Oncology, Department of Internal Medicine, Kangnam Sacred-Heart Hospital, Hallym University Medical Center, Hallym University College of Medicine, Seoul, Republic of Korea

**Keywords:** non-small-cell lung cancer, immune checkpoint inhibitor, squamous, non-squamous, meta-analysis

## Abstract

Immune checkpoint inhibitors (ICIs) have been approved for patients with advanced non-small-cell lung cancer (NSCLC), regardless of histology. However, histologic subtypes of NSCLC may influence treatment outcomes of ICIs. We conducted this meta-analysis to investigate if there is difference in survival benefits of ICIs between squamous (SQ) and non-squamous (non-SQ) NSCLC. We searched PubMed, MEDLINE, EMBASE and ESMO databases. We included randomized controlled trials with the data of survival outcomes in advanced NSCLC patients treated with ICIs. From 7 eligible studies, 998 patients with SQ NSCLC and 2,769 with non-SQ NSCLC were included in the meta-analysis. ICIs improved progression-free survival (PFS) significantly in patients with SQ NSCLC (HR = 0.68 [95% confidence interval (CI), 0.51-0.91], *P* = 0.01), compared to chemotherapy. For patients with non-SQ NSCLC, however, ICIs were not associated with significant improvement of PFS (HR = 0.88 [95% CI, 0.67-1.16], P = 0.37). In terms of overall survival (OS), ICIs prolonged OS significantly in both SQ (HR = 0.71 [95% CI, 0.60-0.83], *P* < 0.0001) and non-SQ NSCLC (HR = 0.77 [95% CI, 0.63-0.94], *P* = 0.01). In conclusion, this meta-analysis indicates that ICIs significantly prolong OS in both SQ and non-SQ NSCLC.

## INTRODUCTION

Recently, immune checkpoint inhibitors (ICIs) have emerged as a new therapeutic option for patients with advanced non-small-cell lung cancer (NSCLC). The programmed death 1 (PD-1) receptors expressed on activated T-cells are activated by the programmed death-ligand 1 (PD-L1) and PD-L2 on tumor cells. The interaction of PD-1 with PD-L1 and PD-L2 promotes tumor immune escape by downregulating T-cell activation [1, 2]. Anti-PD-1/PD-L1 therapy refers to ICI antibodies to block PD-1/PD-L1-mediated inhibitory signals and restore antitumor immunity. A number of randomized trials among all NSCLC subtypes have demonstrated that ICIs (nivolumab, pembrolizumab, and atezolizumab) showed superior outcomes compared to chemotherapy [3–8].

Although ICIs showed survival benefit in patients with advanced NSCLC, there is a great need to identify candidates who will respond most likely to ICIs. Many studies showed the correlation between the efficacy of ICIs and PD-L1 expression on tumor cells and/or tumor-infiltrating immune cells [3, 5, 6]. Because patients with PD-L1-negative NSCLC could also benefit from ICIs [8], however, the predictive value of PD-L1 expression is still controversial [4, 9]. Mutational load may be another possible marker of response to ICIs in NSCLC [10, 11]. Subgroup analysis of clinical trials with ICIs in advanced NSCLC showed that smoking history was associated with improved survival outcome [4, 7]. Smoking would be associated with more mutational load, which might make tumors more immunogenic. Causal relationship between cigarette smoking and lung cancer has been well known, with the stronger association with squamous (SQ) cell carcinoma than adenocarcinoma [12].

Therefore, it can be presumed that histologic subtypes of NSCLC influence treatment outcomes of ICIs. We conducted this meta-analysis of randomized clinical studies to investigate whether there is difference in the survival benefits of ICIs between SQ and non-SQ NSCLC.

## RESULTS

### Results of search

**Figure 1:**
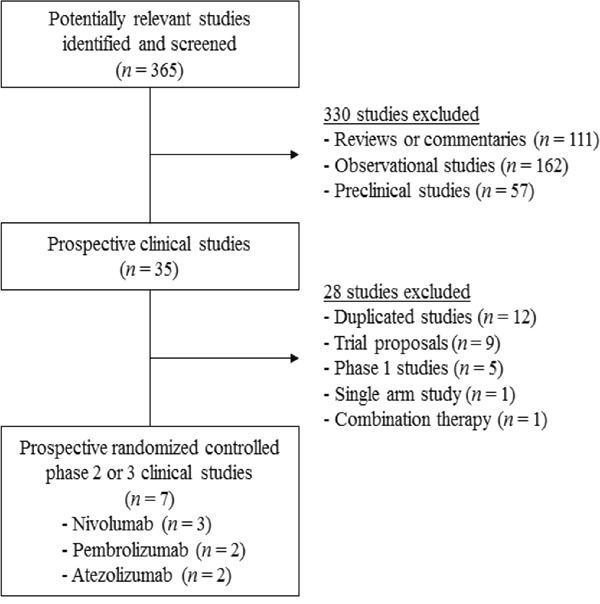
Flow diagram of search process

Figure 1 shows the flowchart of studies through the selection process. A total of 365 studies were identified according to the searching strategy; 330 were excluded after screening the titles and abstracts. Out of the remaining 35 potentially relevant prospective studies, 28 studies were excluded according to the inclusion criteria. One randomized phase 2 trial investigating the adding effect of ICI to chemotherapy as first-line treatment were also excluded [13]. Finally, 7 randomized controlled phase 2 or 3 clinical trials were included in the meta-analysis [3–9].

### Characteristics of the eligible studies

Among 7 eligible studies, one (CheckMate-017) was conducted in patients with SQ NSCLC [3] and another (CheckMate-057) in those with non-SQ NSCLC [4]. In one study (KEYNOTE-010) [5], subgroup analysis was performed between SQ cell carcinoma and adenocarcinoma, so we regarded adenocarcinoma as non-SQ NSCLC. The remaining 4 studies were conducted in all subtypes of NSCLC and performed subgroup analysis according to the histologic type (SQ or non-SQ) [5–9]. Finally, the meta-analysis included 998 patients with SQ NSCLC and 2,769 with non-SQ NSCLC.

Table 1 summarizes the characteristics and survival outcomes of the included studies. ICIs used in the enrolled studies included two anti-PD-1 antibodies (nivolumab and pembrolizumab) and one anti-PD-L1 antibody (atezolizumab). Except for 2 studies conducted in first-line setting [7, 9], 5 enrolled patients with previously treated NSCLC [3–6, 8].

**Table 1:** Summary of the 7 eligible studies evaluating the efficacy of immune checkpoint inhibitors versus chemotherapy in advanced non-small-cell lung cancer

Author,study name(year)	Phase	Setting	Histology	PD-L1 cut-off	Treatment	No. ofpatients	HR for PFS(95% CI)	HR for OS(95% CI)
Brahmer *et al*.,CheckMate-017 (2015)	3	2^nd^-line	Squamous	Any	Nivolumab 3 mg/kg q 2 weeks vs.docetaxel	272	0.62 (0.47-0.81)	0.59 (0.44-0.79)
Borghaei *et al*.,CheckMate-057 (2015)	3	2^nd^-line	Non-squamous	Any	Nivolumab 3 mg/kg q 2 weeks vs.docetaxel	582	0.92 (0.77-1.11)	0.73 (0.59-0.89)
Herbst *et al*.,KEYNOTE-010 (2016)	2/3	≥ 2^nd^-line	Squamous	≥1%	Pembrolizumab 2 mg/kg q 3 weeksvs. pembrolizumab10 mg/kgq 3 weeks vs. docetaxel	222	0.86 (0.62-1.20)	0.74 (0.50-1.09)
			Non-squamous	≥1%	Pembrolizumab 2 mg/kg q 3 weeksvs. pembrolizumab 10 mg/kgq 3 weeks vs. docetaxel	708	0.86 (0.71-1.03)	0.63 (0.50-0.79)
Fehrenbacher *et al*.,POPLAR (2016)	2	2^nd^ or3^rd^-line	Squamous	Any	Atezolizumab 1200 mg q 3 weeksvs. docetaxel	97	NA	0.80 (0.49-1.30)
			Non-squamous	Any	Atezolizumab 1200 mg q 3 weeksvs. docetaxel	190	NA	0.69 (0.47-1.01)
Reck *et al*.,KEYNOTE-024 (2016)	3	1^st^-line	Squamous	≥50%	Pembrolizumab 200 mg q 3 weeksvs. platinum-based chemotherapy	56	0.35 (0.17-0.71)	NA
			Non-squamous	≥50%	Pembrolizumab 200 mg q 3 weeksvs. platinum-based chemotherapy	249	0.55 (0.39-0.76)	NA
Socinski *et al*.,CheckMate-026 (2016)	3	1^st^-line	Squamous	≥1%	Nivolumab 3 mg/kg q 2 weeks vs.chemotherapy	129	0.83 (0.54-1.26)	0.82 (0.54-1.24)
			Non-squamous	≥1%	Nivolumab 3 mg/kg q 2 weeks vs.chemotherapy	412	1.29 (1.02-1.63)	1.17 (0.91-1.52)
Barlesi *et al*.,OAK (2016)	3	2^nd^ or3^rd^ line	Squamous	Any	Atezolizumab 1200 mg q 3 weeksvs. docetaxel	222	NA	0.73 (0.54-0.98)
			Non-squamous	Any	Atezolizumab 1200 mg q 3 weeksvs. docetaxel	628	NA	0.73 (0.60-0.89)

Abbreviations: PD-L1, programmed death-ligand 1; HR, hazard ratio; PFS, progression-free survival; OS, overall survival; CI, confidence interval; NA, not available.

### Progression-free survival

**Figure 2:**
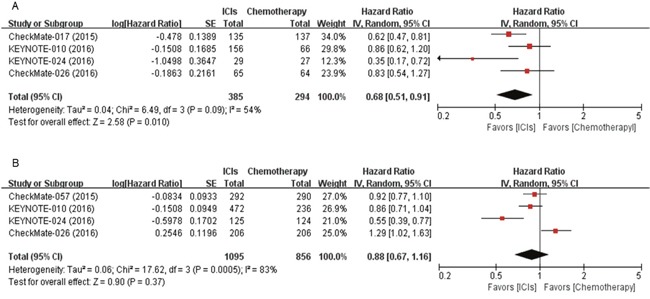
Forest plots of hazard ratios comparing progression-free survival of immune checkpoint inhibitor versus chemotherapy in **(A)** squamous and **(B)** non-squamous non-small-cell lung cancer. ICIs, immune checkpoint inhibitors.

From 5 studies [3–5, 7, 9], 679 patients with SQ NSCLC and 1,951 with non-SQ NSCLC were included in the meta-analyses of hazard ratios (HRs) for progression-free survival (PFS) (Figure 2). ICIs, compared with chemotherapy, improved PFS significantly in patients with SQ NSCLC (HR = 0.68 [95% confidence interval (CI), 0.51-0.91], *P* = 0.01) (Figure 2A). We adopted random effect model because there was significant heterogeneity (*X*^2^ = 6.49, *P* = 0.09, *I^2^* = 54%). For patients with non-SQ NSCLC, ICIs were not associated with significant improvement of PFS (HR = 0.88 [95% CI, 0.67-1.16], *P* = 0.37) (Figure 2B). We also applied random effect model because significant heterogeneity was observed (*X*^2^ = 17.62, *P* = 0.0005, *I^2^* = 83%).

### Overall survival

**Figure 3:**
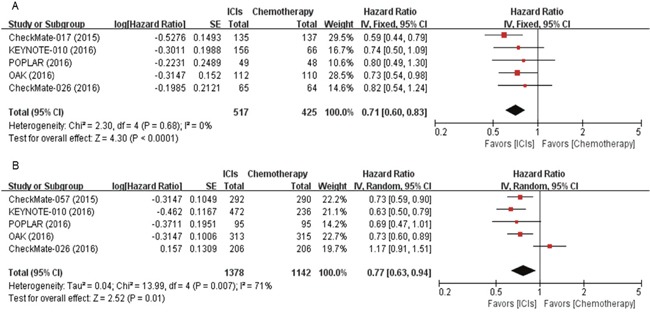
Forest plots of hazard ratios comparing overall survival of immune checkpoint inhibitor versus chemotherapy in **(A)** squamous and **(B)** non-squamous non-small-cell lung cancer. ICIs, immune checkpoint inhibitors.

Six studies with 942 SQ NSCLC patients and 2,520 non-SQ NSCLC cases reported HRs and 95% CIs for overall survival (OS) [3–6, 8, 9]. After the meta-analysis, we found that ICIs induced 29% reduction of the death risk in patients with SQ NSCLC (HR = 0.71 [95% CI, 0.60-0.83], *P* < 0.0001) (Figure 3A). There was no significant heterogeneity (*X*^2^ = 2.30, *P* = 0.68, *I^2^* = 0%). For patients with non-SQ NSCLC, ICIs also induced 23% reduction in the risk for death (HR = 0.77 [95% CI, 0.63-0.94], *P* = 0.01) (Figure 3B). Random effect model was used because there was significant heterogeneity (*X*^2^ = 13.99, P = 0.007, *I^2^* = 71%).

## DISCUSSION

We conducted this study to investigate whether the survival benefits of ICIs is different between histologic subtypes (SQ versus non-SQ) of advanced NSCLC. The meta-analysis of 7 relevant studies demonstrated that ICIs, compared to chemotherapy, showed better survival in both SQ and non-SQ NSCLC patients.

Recent whole exome sequencing study demonstrated a significant correlation between the total mutation load and clinical benefit with ICIs in NSCLC [10]. Therefore, mutational load may be a possible marker of response to ICIs. Mutational profiles are significantly different between SQ cell carcinoma and adenocarcinoma in NSCLC [10, 14, 15]. In addition, it is well known that smoking is linked to the expression of neoantigens and increased numbers of somatic mutations. Smoking is more frequently associated with SQ than non-SQ NSCLC [12]. Thus, we assumed that histologic subtypes of NSCLC might influence the survival outcomes of ICIs. In this meta-analysis, although ICIs failed to improve PFS significantly in patients with non-SQ NSCLC, they prolonged OS in both SQ and non-SQ NSCLC, compared to chemotherapy. The survival benefit from ICIs regardless of histologic subtypes in patients with advanced NSCLC may have several explanations. First, the difference in the mutational burden between SQ and non-SQ NSCLC might not be significant. Second, other biomarkers including PD-L1 expression level might interact to dilute the effect of difference in the mutational load. Third, frontline treatment may influence the effect of the subsequent immunotherapy. It has been reported that chemotherapy changes the immune microenvironment of tumor in various way [16] and dynamically alter the PD-L1 expression on tumor cells [17, 18]. Of 7 studies included in the meta-analysis, 5 had been conducted in more than second-line setting. Lastly, the different rates of KRAS mutation between SQ NSCLC and non-SQ NSCLC may affect the survival results. KRAS mutations in NSCLC are detected more frequently in adenocarcinoma than SQ cell carcinoma [19]. Subgroup analysis in the CheckMate-057 trial showed that patients with KRAS mutation were more likely to benefit from nivolumab in terms of an improved OS [4].

In general, patients with PD-L1 expression-positive tumor show better outcomes from ICIs, compared to those with PD-L1 expression-negative tumor [4, 6]. The low level of PD-L1 expression of tumors may be one of the plausible explanations for the failure in the CheckMate-026 [9]. This study enrolled a broad range of patients with low PD-L1 expression threshold of just ≥ 1%. This threshold level was much lower than that (≥ 50%) of successful KEYNOTE-024 trial with pembrolizumab [7]. Although PD-L1 expression is not a perfect biomarker, therefore, it seems that high-level PD-L1 expression is a useful predictor for the effect of ICIs. The impact of PD-L1 expression level on survival outcomes may be more critical in patients with non-SQ NSCLC than those with SQ NSCLC. Survival benefit of nivolumab was independent of PD-L1 expression levels in the SQ NSCLC trial (CheckMate-017) [3], contrast to the non-SQ NSCLC trail (CheckMate-057) in which OS benefit correlated with PD-L1 expression level [4]. In non-SQ NSCLC, therefore, patients with high PD-L1 expression may have greater benefit from ICIs than those with PD-L1-negative or weak expression. The role of PD-L1 expression needs to be urgently revealed to guide the optimal use of ICIs in NSCLC.

Of note, our study has several potential limitations. First, this meta-analysis included heterogeneous studies conducted in different treatment settings with various levels of PD-L1 expression. Second, there was significant heterogeneity among studies. Although we used random effect model to minimize its influence on the results, the pooled HRs might be affected by the heterogeneity. Third, because the small number of studies was currently available, we could not analyze the survival according to the treatment setting (first-line or more than second-line). In addition, we could not compare survival benefits in the subgroups according to the PD-L1 status because of the limited data.

In conclusion, our meta-analysis indicates that ICIs significantly prolonged OS in both SQ and non-SQ NSCLC compared to chemotherapy. Since this meta-analysis included heterogeneous clinical trials with different treatment settings and various levels of PD-L1 expression, however, further studies are needed to evaluate the impact of histology on the effect of ICIs in patients with advanced NSCLC.

## MATERIALS AND METHODS

### Searching strategy

We carried out a systematic search of PubMed, MEDLINE, and EMBASE from January 2000 to November 2016. We also searched abstracts and virtual meeting presentations from the ESMO 2016 Congress. The following searching terms were used: ‘immune checkpoint inhibitor or immunotherapy’, ‘nivolumab or pembrolizumab or atezolizumab or ipilimumab or tremelimumab’, ‘advanced or metastatic’, ‘non-small-cell lung cancer or lung cancer or NSCLC’. All eligible studies were retrieved and their bibliographies were checked for other relevant publications. When the data were unclear or incomplete, the corresponding author was contacted to clarify data extraction.

Eligible studies were required to meet the following inclusion criteria: prospective randomized controlled phase 2 or 3 trials in patients with NSCLC; randomization of patients to treatment with either ICI monotherapy or chemotherapy; performing subgroup comparison of PFS or OS by the histology (SQ or non-SQ); providing HR and its 95% CI for PFS or OS.

### Data extraction

The following data were carefully extracted from all eligible studies: first author's name, year of publication, trial phase, number of patients, treatment setting and regimen, PD-L1 expression level, PFS and OS to ICIs stratified by histology and their HR with 95% CI.

Data extraction was done independently by two of the authors (BJK and HSK). If these two authors could not reach a consensus, another author (JHK) was consulted to resolve the dispute.

### Statistical analyses

The aim of this meta-analysis was to compare survival outcomes (OS and PFS) between SQ and non-SQ NSCLC treated with ICIs. Statistical values used in the analysis were obtained directly from the original article or abstract and heterogeneity between studies was estimated using the *I*^2^ inconsistency test and chi-square-based Cochran's *Q* statistic test in which *P* < 0.1 was taken to indicate the presence of significant heterogeneity. A fixed effect model (Mantel-Haenszel method) was used to calculate the pooled HRs when substantial heterogeneity was not observed. When substantial heterogeneity was observed, we applied a random effects model (DerSimonian-Laird method). Final results were presented with HRs with 95% its. All reported *P*-values were from two-sided versions of the respective test; *P* < 0.05 was considered statistically significant. RevMan version 5.2 software was used to report outcomes.
